# Deciphering gene mutations in the efficacy and toxicity of antineoplastic drugs: an oncology pharmacist’s perspective

**DOI:** 10.3389/fphar.2025.1574010

**Published:** 2025-03-20

**Authors:** Yucai Jiang, Guolin Alexander Wen

**Affiliations:** ^1^ Department of Pharmacy, Affiliated Hospital of Putian University, Putian, Fujian, China; ^2^ Department of Pharmacy, Dana-Farber Cancer Institute, Boston, MA, United States; ^3^ Faculty of Synapse Program, Massachusetts General Hospital, Harvard Medical School, Boston, MA, United States

**Keywords:** pharmacogenomics, cell-free DNA, circulating tumor DNA, next-generation sequencing, precision medicine, tumor-agnostic drug

## Abstract

**Background/Objectives:**

This article reviews some key emerging pharmacogenomic topics in oncology pharmacy practice.

**Methods:**

Publications selected to review were mainly sourced from the new drug approvals by the Food and Drug Administration and the new regimens listed in the National Comprehensive Cancer Network.

**Results:**

Key pharmacogenomic topics were presented, including genetic alterations influencing drug metabolism, drug efficacy, and changes in therapeutic targeting; Relevant clinical updates and advancements were summarized to provide an in-depth understanding.

**Conclusion:**

The abundance of pharmacogenomic measures builds a solid foundation and heralds a paradigm shift toward individualized patient care.

## 1 Overview

Pharmacogenomics, a scientific term coined in 1997, is the study of drugs responding to individual genetic alterations, i.e., mutations, gene expression regulation, and changes in alleles. Recent decades have seen a surge in pharmacogenetic findings. Current pharmacogenomic topics are spotlighted in multidrug-resistant genes, drug metabolism polymorphisms, and oncology precision medicine.

The pharmacogenomic field paved the way for tailoring drug therapies based on genetic profiles. The American Society of Clinical Oncology published its “Somatic Genomic Testing in Patients with Metastatic or Advanced Cancer: Provisional Clinical Opinion” in 2022. This provides guidance on when next-generation sequencing should be performed, what type of assay should be performed, and then how these results should guide treatment decisions. It also lists several definitions that are commonly used in precision oncology (Chakravarty et al., 2022). This will continue to serve as a cornerstone for precision oncology practice.

Understanding the complexities of molecular testing is pivotal in oncology practice, ensuring tailored treatment strategies for improved patient outcomes. Gene-drug pair data are widely published and applied in precision oncology. Epidermal growth factor receptor (*EGFR*)exon 19 deletion carriers of non-small cell lung cancer have so-called sensitized clonal lesions that respond well to tyrosine kinase inhibitors I.E. osimertinib, furmonertinib, and aumolertinib (Li et al., 2022). This acts as a good example of gene-drug pairs in precision oncology. Additionally, gene-drug pairs crucial for drug metabolism merit attention in clinical practice. This is exemplified by dihydro-pyrimidine dehydrogenase (*DPyD*) wild-type gene carriers that have normal metabolism of capecitabine, tegafur, and 5-fluorouracil (Dean et al., 2012).

Owing to the complexity and evolutionary nature of this subject, a pharmacogenomics review is of great importance and high priority. Ongoing advancements in genomic sequencing technologies continue to refine our ability to identify actionable genomic alterations with higher precision and efficiency. Consequently, the integration of genomic information into clinical practice heralds a paradigm shift towards more effective and individualized approaches to patient care.

This review is aimed at supplying an evidence-based reference in pharmacogenomics for cancer treating clinicians. Incorporating pharmacogenomic insights into oncology practice will assist treatment selection approaches, which in turn armors healthcare providers with drug and dosing personalization as per different genetic profiles. Genetic variations have an interplay with drug efficacy and toxicity, with which the appropriate utilization of these knowledge-based skills improves survival outcomes while minimizing adverse effects.

This review serves as a guide for oncologists, pharmacists, and nurses pursuing the pharmacogenomic impacts within the scope of the clinical decision-making process in patient care. It also introduces a limited scope of certain blockbuster drugs and the scientific foundations behind their approvals, aiming to provide an update for managed care services when financial decisions must be made.

## 2 Understanding cell-free DNA assays

Building a pharmacogenetic and pharmacogenomic arcade necessitates using fundamental toolkits to conduct molecular testing: sequencing, immunohistochemistry, flow cytometry, single cell analysis, etc. Inevitably, they all have pros and cons; however, one testing stands out from the crowd: cell-free DNA (cfDNA) assays. Compared to bricks and stones, cfDNA assays are a “supercement”-type toolkit that enables high-input, high-output DNA testing. The cfDNA assays are capable of testing millions of gene mutations simultaneously at one single test with a turnaround time of 5–14 days. This cfDNA toolkit made a revolution in the pharmacogenetic study, shortens testing time multifold, and plays a critical role in maximizing all subsequent processes, i.e., result interpretation, treatment selection, making clinical decisions, and final patient outcome.

Cell-free DNA (cfDNA) testing, also known as liquid biopsy, is a non-invasive method used to analyze fragments of DNA that are circulating freely in the bloodstream. These DNA fragments are released by cells throughout the body, including tumor cells, and can provide valuable information about genetic mutations, chromosomal abnormalities, and other molecular markers associated with various health conditions, such as cancer. In oncology care, it is warranted due to progressive disease in multiple metastatic sites, and it demonstrates progressive mutations other than those originally discovered in primary cancer.

The cell-free DNA assay and the circulating tumor DNA (ctDNA) assay, despite a terminological difference between them, are often used interchangeably in practice.

The cfDNA assay, being a nuclear weapon for geneticists, has a variety of calipers. Years of experience have given onco-pathologists different choices: whole exome sequencing, targeted panel sequencing, whole genome sequencing, ultra-deep sequencing (Van Dessel et al., 2019), with all the terms being self-explanatory. Next-generation sequencing (NGS) nowadays serves as a broad-spectrum term that encompasses various sequencing methods and other similar-sounding terms. Testing of epigenetic features is made available, such as methylation-based sequencing (Rauluseviciute et al., 2019), phosphorylation-based testing (Yu et al., 2025), ribosome sequencing, etc.

Difficulties have been encountered by oncology clinicians, and one of the major challenges is the instability and unique characteristics of RNA samples, rendering a dependence on the RNA sequencing (Kukurba and Montgomery, 2015). RNA-sequencing helps detect transcriptomics, fusion genes, alternative splicing, and expression levels due to the inability of DNA sequencing methods. There is also a challenge when Minimal Residual Disease (MRD) detection is needed to identify rare cancer cells in the blood. In such cases, ultra-deep technology might be the best ammunition for pinpointing at a tiny “pixel” in the atlas. Ultra-deep sequencing refers to sequencing a DNA/RNA region at extremely high depth (e.g., 10,000× or more coverage) to detect low-frequency variants or rare mutations (van Dessel et al., 2019). NGS is the underlying technology that enables ultra-deep sequencing by generating millions to billions of reads in parallel.

Germline noise, which geneticists could also face, is defined as germline gene variations disturbing a limited number of somatic alterations and causing background interference for the latter. A majority of the commercial assays do not implement this strategy, but rather report germline alterations as variants of uncertain significance. However, assays could analyze and match tumors against normal tissues, and subtract out for the germline noise (Koboldt et al., 2013).

Several commercial cell-free DNA assays are available and being incorporated in oncology practice. These assays are to be utilized in more advanced cancer diseases seeking actionable mutations and acquired resistances. Additionally, these assays are used in testing minimal residual disease (MRD) of chronic myeloid leukemia and explored in metastatic/advanced colorectal cancer (Jacome and Johnson, 2023).

Patients exhibiting positive responses to therapy may yield false negative test results. Therefore, precise timing of testing becomes imperative, particularly in scenarios involving minimal disease burden or ongoing response to treatment. In such instances, the absence of cells actively releasing circulating tumor DNA could potentially lead to misleading negative test outcomes.

Even though tissue biopsy has been used for decades in testing genomic variants, challenges posed include being an invasive procedure, lacking accessibility to intracranial lesions, and being decalcified of osseous lesions. Moreover, the biggest challenge is tumor heterogeneity in different spaces and different stages, for instance, a primary tumor originates in the lung and subsequently metastasizes to the liver, adrenal glands, and brain, potentially harboring identical driver mutations across these distant sites. As the tumor undergoes evolutionary progression within these disparate microenvironments, distinct mutational profiles may emerge after unequal responses to therapeutic interventions. Consequently, the genetic landscape of distant lesions may exhibit marked heterogeneity, reflecting the dynamic interplay between tumor evolution and therapeutic selection pressures (de Visser and Joyce, 2023).

Spatial isolation is another cause of tumor heterogeneity. Distant metastases may bear a different histology under the microscope, which has been proved correct under many circumstances. A good example of evidence is colorectal cancer cases, when there is hepatic metastasis, often have different pathological features from the primary location and hence become less responsive to pyrimidine-platinum-based systemic therapy. Local ablation, embolization, or radiation therapies are options to treat hepatic lesions (Halama et al., 2013).

Clonal evolution refers to the progression of a tumor from primary pathological clones to new clones, often resistant to ongoing therapy. This resistant clone is not necessarily created *de novo* but typically originates from an indolent precursor. As a result, heterogeneous clones may coexist while undergoing antineoplastic treatment. A notable example is lung cancer, where, after treatment with EGFR-targeted tyrosine kinase inhibitors (TKIs), some clones become highly resistant to TKIs, poised for proliferation when given the opportunity (Roper et al., 2020).

The cell-free DNA assays overcome heterogeneity by picking up a DNA mixture sourced from different lesions to better represent tumor heterogeneity. One drawback of the cell-free DNA assays is that “hermit” lesions might be unable to release DNA debris to the main circulation, I.E. from brain metastasis.

There are numerous commercial assays for cell-free DNA. A credentialed methodology might be pursued because all commercial assays are not deemed the same, but rather differ in key characteristics based on the qualitative and quantitative features of gene chips. The testing of MRD falls under the category of qualitative cell-free DNA assays. On the other hand, quantitative cell-free DNA assays evolve and seek the volume of mutation, I.E. copy number; this is incredibly important for assessing acquired resistance, mutations, and changes in allele frequency. The cell-free DNA assays generally implement both of these functionalities.

Two critical terms in the world of diagnostic testing are “companion” and “complementary.” Companion testing serves as a guiding light, ensuring the safe and effective use of a related medication. Its presence significantly enhances the risk-benefit ratio of the treatment. For instance, the BRAF assay for encorafenib initiation in melanoma patients is invaluable for treatment decisions. The difference between the two terms is that “companion” generally indicates a Food and Drug Administration (FDA) requirement, while the term “complementary,” being self-explanatory, is merely a suggestion.

## 3 A journey from mutation to action

The result of genomic testing is used to further refine current/subsequent lines of therapy. Clinical actionability is defined as a clinical decision being made from a genetic alteration that predicts a response/resistance/toxicity to a particular therapy. This includes conferring beneficial/toxic outcomes of a specific drug, identifying appropriate drugs/dosage in the patient’s tumor type as well as seeking specific clinical trials for this particular alteration. The cell-free DNA assays are used, I.E. in the lung cancer realm, to see what acquired mutations are after disease progression on a targeted therapy line.

### 3.1 Gene polymorphism in pharmacokinetics and pharmacodynamics

#### 3.1.1 Dihydropyrimidine dehydrogenase gene (*DPyD* gene)

The *DPyD* gene encodes dihydropyrimidine dehydrogenase (DPD), a key enzyme involved in the metabolism of fluoropyrimidine drugs such as 5-fluorouracil (5-FU) and capecitabine. DPD plays a crucial role in the catabolism of these drugs, converting them into inactive metabolites. Genetic variations in the *DPyD* gene can lead to reduced DPD enzyme activity, resulting in impaired drug metabolism and increased systemic exposure to active fluoropyrimidine metabolites. This can predispose individuals to severe and potentially fatal toxicities, such as 5-FU-associated toxicities, highlighting the importance of DPD testing in optimizing fluoropyrimidine-based chemotherapy regimens to ensure both efficacy and safety for cancer patients. This DPD enzyme also metabolizes tegafur (approved in other countries but not by the FDA) as it is a prodrug of 5-fluorouracil and DPD deficiency could lead to 5-FU-associated toxicities (Leung, 2017).

#### 3.1.2 Uridine diphosphate glucuronosyltransferase 1A1 (*UGT1A1*)

The *UGT1A1* enzyme is encoded by the *UGT1A1* gene, which is of central significance in the metabolism of various endogenous compounds and xenobiotics, including bilirubin and drugs. Specifically, *UGT1A1* is responsible for the glucuronidation of bilirubin, a process essential for its excretion from the body. Genetic variations in the *UGT1A1* gene, such as the *28/*28 homozygous and *6/*28 heterozygous genotypes can lead to reduced enzyme activity, resulting in impaired govitecan conjugation and increased toxic events of govitecan (Rozenblit and Lustberg, 2022). This can also impact the metabolism of other drugs that are substrates for *UGT1A1*, including irinotecan and etoposide, potentially increasing the risk of toxicity (Negoro et al., 2019). Enasidenib, acting both as a substrate and inhibitor of *UGT1A1*, is associated with hyperbilirubinemia due to off-target inhibition of the *UGT1A1* enzyme (14% overall; 8% grade 3–4) (DiNardo et al., 2023). Therefore, understanding the genetic variability in the UGT1A1 gene is crucial for personalized medicine approaches, particularly in the context of drug metabolism and individualized dosing strategies.

#### 3.1.3 O-6-methylguanine-DNA methyltransferase gene (*MGMT* gene)

The *MGMT* is an instrumental enzyme in repairing DNA damage. *MGMT’s* repair work is vital for normal cellular processes, and its functionality, by enabling direct reversal of alkylation, can lead cancer cells to develop resistance to chemotherapeutic alkylators. Examples are temozolomide and dacarbazine, which are alkylators frequently employed in the management of brain tumors, sarcoma, and lymphoma. The efficacy of these drugs can be compromised by the presence of *MGMT* in cancer cells (Wu et al., 2021). This predictive biomarker role of *MGMT* methylation was confirmed in the Stupp trial. A significantly larger survival benefit was reported from adding temozolomide to standard radiotherapy in *MGMT*-methylated glioblastomas compared to the *MGMT*-unmethylated group (Hegi et al., 2005). Subsequent long-term outcomes have been reported, affirming the persistent significance of *MGMT* gene promoter methylation in predicting temozolomide sensitivity. *MGMT* promoter methylation in glioblastoma is both a prognostic marker and a predictive one for response to treatment with alkylating agents (Chen and Wen, 2024).

#### 3.1.4 Thiopurine S-methyltransferase (*TPMT*) gene

The *TPMT* gene encodes the *TPMT* enzyme, which is pivotal in metabolizing thiopurine drugs used in the treatment of various conditions such as autoimmune diseases and leukemia. Thiopurine drugs, including azathioprine, 6-mercaptopurine, and thioguanine, are converted into active metabolites through *TPMT*-mediated methylation. However, individuals with certain genetic variants of the *TPMT* gene may have reduced enzyme activity, leading to altered drug metabolism and increased risk of adverse reactions, such as myelosuppression. This exemplifies a clinically significant drug-gene interaction, where variations in the *TPMT* gene influence the efficacy and safety of thiopurine medications, necessitating personalized dosing regimens and close monitoring to optimize therapeutic outcomes while minimizing potential side effects.

#### 3.1.5 Cytochrome P450 (CYP) isoenzymes

The CYP isoenzymes play a critical role in the oxidative metabolism of therapeutic substances, representing a major pathway for drug metabolism. With 58 different human CYP genes identified through genome sequencing, various CYP isoenzymes are encoded. Environmental factors, including drug-drug interactions, can influence CYP enzyme activity, such as the induction or inhibition of CYP3A4, altering the metabolism of other drugs. Pharmacogenomic relevance lies in the polymorphic nature of many CYP genes involved in xenobiotic metabolism, affecting drug metabolism in a significant portion of the population, with prevalence varying by ancestry. The polymorphisms can lead to varied metabolic phenotypes, including ultra-rapid metabolizers, extensive metabolizers, and poor metabolizers. Notably, clinically significant polymorphic variations are observed in genes such as CYP2C9, CYP2C19, CYP2D6, and CYP3A4, responsible for metabolizing a majority of therapeutic medications (von Moltke et al., 1998).

Drug-gene interactions that influence drug metabolism may involve multiple genes. Irinotecan is a notable example of a drug with polygenic interactions based on current evidence. Irinotecan is widely used in the treatment of lung cancer, gastrointestinal cancer, and ovarian cancer, etc. Irinotecan undergoes primarily hepatic metabolism, converting to its active metabolite govitecan via carboxylesterase enzymes. Additionally, it may undergo CYP3A4-mediated oxidative metabolism to form several inactive metabolites, one of which can be hydrolyzed to release govitecan. When all three phenotypes, CYP3A4, *UGT1A1* (as discussed in an earlier chapter) and carboxylesterase are taken into consideration, the resulting clinical scenarios for polygenic drug interaction become increasingly complex. These should be intervened through a case by case analysis.

#### 3.1.6 Polygenic risk scores

Over the past decades, scientists and oncologists have discovered many gene mutations as drug targets. For example, the rat sarcoma viral oncogene homolog (*RAS*) mutation has been studied for more than half a century before recently becoming a targetable mutation, whereas a new ten eleven translocation 2 (*TET2*) mutation emerged as a potential target last year (Zou et al., 2024). In contrast, mutations like tumor protein p53 (*TP53*) have not seen breakthroughs in becoming drug targets. Predicting whether a mutation could be a drug target is extremely difficult.

Given this, drug researchers today can approach the issue from a different standpoint by considering multiple mutations in combination. Hence, a polygenic risk scoring scale could be tested, analyzed, and utilized to support drug approvals. A successful case study is olaparib, which has been granted FDA approval for prostate cancer subsequent therapy and ovarian cancer maintenance therapy. The prostate cancer approval is based on homologous recombination repair (HRR) gene mutations (Scott et al., 2021), whereas the ovarian cancer indication is supported by homologous recombination deficiency (HRD) testing, which is similar to that used for prostate cancer (Konstantinopoulos et al., 2015).

Another successful case study is the use of polygenic risk scores to predict the benefits of chemotherapy for early breast cancer patients with estrogen receptor-positive, HER2 (human epidermal growth factor receptor 2)-negative pathology. An Oncotype DX score set at a threshold of 25 is a strong predictor and is listed in the National Comprehensive Cancer Network (NCCN) guidelines. Ongoing efforts are being made to implement predictive multigenic scores in the treatment and screening of other diseases, such as lung cancer and colorectal cancer, with the hope of achieving positive trial outcomes and benefiting patients.

### 3.2 Tumor resistance and genes

Tumor resistance involves various mechanisms that contribute to the survival and growth of cancer cells. Genetic mutations on drug targets reduce drug binding (e.g., EGFR mutations in lung cancer resist TKIs like erlotinib) and avoid cytotoxic attacks, enabling continued proliferation. Cancer stem cells play a significant role, yielding tumor plasticity, as then tumors have the ability to evolve to a different histology. These mechanisms help tumors resist therapy. Additionally, DNA damage repair, homologous recombination (HR) or nucleotide excision repair (NER), can restore genes allowing them to escape apoptosis. Gene polymorphism and mutations play a role in resistance mechanisms; as elaborated hereinafter are several notable examples of monogenic mutations and polygenic mechanisms.

#### 3.2.1 Alternative pathways and off-target mutations

Cancer cells activate alternative signaling networks when a specific pathway is dysfunctionally blocked. For instance, consistently inhibiting *EGFR* can have cancer cells activate alternative growth pathways such as HER2 or *MET* (mesenchymal-epithelial transition factor) to continue growth. Off-target mutation occurs in these circumstances. A mutation in *KRAS* or *BRAF* can render certain targeted therapies ineffective, as these mutations can activate downstream pathways that promote cell survival and resistance to the drug, even if the drug is not directly targeting these mutations.

#### 3.2.2 ATP-binding cassette G2 subfamily (*ABCG2*) gene and efflux pump

Efflux pumps can transport drugs extracellularly from tumor cells. Higher efflux pump function is a mechanism of resistance discovered in some chemotherapeutic treating courses. The breast cancer resistance protein (BCRP) is an efflux pump protein that transports methotrexate and topotecan (U.S. Food and Drug Administration, 2019). BCRP, encoded by the *ABCG2* gene, is found in the liver, kidney, central nervous system, and gastrointestinal tract. *ABCG2* gene polymorphisms (Heyes et al., 2018) and BCRP-involved drug-drug interactions have raised attention. Hundreds of BCRP inhibitors have been identified (Mao and Unadkat, 2015), including febuxostat, linezolid, lansoprazole, ketoconazole, and other benzimidazoles (Breedveld et al., 2004; Ikemura et al., 2019). As a result, BCRP has now been recognized by the FDA to be one of the key drug transporters involved in clinically relevant drug disposition.

#### 3.2.3 Tumor microenvironment and polygenic score prediction

The Tumor Microenvironment (TME) is the ecosystem that surrounds a tumor inside the body, including immune cells, the extracellular matrix, blood vessels, and other cells like fibroblasts. The tumor and its microenvironment interact and constantly influence each other. This dynamic relationship can contribute to resistance, as seen in adrenal cortical carcinoma, which does not respond to immunotherapy due to low tumor mutational burden, high vascularity, and low or absent PD-L1 expression. However, adding lenvatinib or cabozantinib might alter the microenvironment, potentially inducing PD-L1 positivity and improving responsiveness to therapy (Bedrose et al., 2020).

Mounting an immune response against cancer involves numerous steps, but tumors can counteract these mechanisms. In head and neck squamous cell carcinoma (HNSCC), the tumor microenvironment is characterized by multiple immune suppression mechanisms, necessitating a multipronged therapeutic approach to overcome them. Myeloid-derived suppressor cells (MDSCs) and macrophages interact bidirectionally to enhance immune suppression, a phenomenon observed in both tumors and other conditions. Additionally, regulatory T (Treg) cells contribute to immune evasion by inducing apoptosis of CD8^+^ T cells and inhibiting the proliferation of CD4^+^ T cells (Wang et al., 2019).

TME biomarker development focuses on solid tumors, including triple-negative breast cancer, ovarian cancer, metastatic melanoma, head and neck cancer, and other mixed solid tumors. VIGex, a 12-gene expression profile involving genes related to immune activation and suppression, including CTLA-4 and CD274 (PD-L1), has been explored as a potential predictive biomarker for treatment with immune checkpoint inhibitors. In the INSPIRE trial, the VIGex HOT score was significantly associated with improved progression-free survival and overall survival (Hernando-Calvo et al., 2024).

### 3.3 Genetic alterations, drug targets, and tumor agnostic approvals

Tumor-agnostic approvals represent a groundbreaking approach in cancer treatment, transcending traditional categorizations based on tumor origin and instead focusing on specific genetic biomarkers shared across various cancer types. These approvals target distinct molecular alterations such as tumor mutational burden (TMB), mismatch repair (MMR) deficiencies, microsatellite instability (MSI) status, neurotrophic tyrosine receptor kinase gene (*NTRK*) fusions, rearranged during transfection (*RET*) fusion, and rapidly accelerated fibrosarcoma B-type (*BRAF*) mutations. By homing in on these biomarkers, therapies can effectively target the underlying genetic abnormalities driving cancer growth regardless of where the tumor originated in the body. This approach holds immense promise, offering tailored treatments for patients whose cancers harbor these specific genetic alterations, thereby potentially improving outcomes and expanding treatment options beyond conventional methods tied to specific cancer types.

Tumor-agnostic approvals have revolutionized oncology care by shifting the treatment paradigm towards precision medicine, where therapies are tailored to the unique genetic makeup of an individual’s cancer. Tumor-agnostic approvals have a portfolio of TMB, dMMR, MSI, *NTRK* fusion, *RET* fusion, and *BRAF* mutations; each of which is actionable by giving a specific class of FDA-approved agents.

#### 3.3.1 MMR/MSI

Assessing MMR/MSI status provides crucial insights into a tumor’s immunogenicity and susceptibility to immunotherapy, guiding treatment decisions and potentially enhancing responses. In this ever-evolving landscape, the pursuit of knowledge marches onward. Discussions buzz with anticipation, hinting at potential breakthroughs on the horizon. Amidst the choices, pembrolizumab emerged as the beacon of hope. Pembrolizumab stands tall as the pioneer extending their reach to MSI-high solid tumors (Table 1) along with dostarlimab. Nivolumab is incorporated in the NCCN guidelines for MSI-high solid tumors (with or without ipilimumab, per the NCCN compendium referenced in December 2024).

**TABLE 1:** Pembrolizumab agnostic approvals and specific diagnostic requirements.

Pembrolizumab agnostic approval	Testing suggested	Diagnostic requirement
Tumor mutational burden	Next-generation sequencing (NGS)	≥10^a^
Mismatch repair	Immunohistochemistry (IHC)	Deficient
Microsatellite instability	Polymerase chain reaction (PCR)/NGS	High
*POLE*/*POLD1* ^b^	PCR/NGS	Mutated
PD-L1^c^	Immunohistochemistry	≥50

^a^ This cut-off value is approved with tissue biopsy.

^b^ This NCCN endorsement is for colorectal cancer, small bowel cancer, and appendiceal cancer.

^c^ This indication was authorized in certain countries; Contraindications to pembrolizumab should be screened before prescribing. PD-L1 (programmed death ligand 1).

Patients with deficient MMR or MSI-high status exhibit similar characteristics. Instances of discrepancies may arise, particularly between endometrial and colorectal cancers, which are different in prevalence among germline carriers of lower penetrant genes like *MSH6* (MutS Homolog6) and *PMS2* (postmeiotic segregation increased 2) (Kim et al., 2022). However, generally speaking, deficient MMR indicated by immunohistochemistry (IHC) tests correlate with MSI-high status, and *vice versa*. Colorectal and endometrial cancers are the most common sites for these abnormalities, though they can occur less prevalently in other cancer types such as prostate cancer (∼3%) or gastric cancer.

#### 3.3.2 Tumor mutational burden (TMB)

TMB quantifies non-synonymous mutations, potentially leading to neo-antigen formation. While higher TMB generally correlates with better immunotherapy responses across cancer types, exceptions like primary gliomas exist (Khasraw et al., 2020). Pembrolizumab gained FDA approval via the KEYNOTE-158 trial, showcasing efficacy in tumors with a TMB of 10 mutations per megabase (mut/Mb) (Cristescu et al., 2022). TMB higher than or equal to 10 mut/Mb is generally accepted as actionable in tissue-based testing (Table 1); however, whether cfDNA assay requires a higher actionable TMB value is still debatable. Ongoing research investigates the utility of cell-free DNA analysis in determining an actionable TMB value (Krizova et al., 2022). By embracing tumor-agnostic approaches, oncology enters a new era of personalized medicine, offering hope for improved outcomes and better quality of life for patients with diverse cancer types.

#### 3.3.3 BRAF

Identifying *BRAF* mutations allows for targeted therapies that can effectively inhibit the aberrant signaling pathways driving cancer growth. Currently, there are several approved tyrosine kinase-inhibiting drugs. Dabrafenib and trametinib are now approved for tumors with *BRAF V600E* mutations including glioblastoma and biliary tract cancer (Subbiah et al., 2020; Wen et al., 2022). This approval followed promising results from clinical studies across various tumor types, leading to a tumor-agnostic indication (NCI. *BRAF*) (Winstead, 2022). It is worthwhile mentioning this combination was an off-label use for some non-*BRAF-V600E* mutations in case reports and clinical trials (Shimoi et al., 2024).

#### 3.3.4 *NTRK* fusion

Larotrectinib was initially approved for tumors with *NTRK*-activating fusions, including pediatric cases. Targeting *NTRK* fusions with the specific tyrosine kinase inhibitor entrectinib has resulted in remarkable responses, even across different tumor types (Manea et al., 2022). Notably, larotrectinib was the first tissue-agnostic approval for solid tumors harboring *NTRK* fusions, demonstrating efficacy in various tumor types such as salivary gland tumors and soft tissue sarcomas (Iannantuono et al., 2022). On 13 June 2024, repotrectinib received accelerated approval from the FDA for treating tumor-agnostic *NTRK* fusions (U.S. Food and Drug Administration, 2024). Similarly, entrectinib and repotrectinib, which also inhibit Receptor tyrosine kinase 1 ROS1, are approved for *ROS1*-mutated non-small cell lung cancer; however, it is not approved under tumor-agnostic indications.

#### 3.3.5 *RET* fusion

Selpercatnib, approved for tumors with *RET*-activating mutations, has shown efficacy in medullary thyroid cancer and other solid tumors harboring *RET* fusions, as demonstrated in the Libretto trial. These tumor-agnostic approvals represent significant advancements in precision oncology, offering targeted therapies to patients based on specific genetic alterations rather than tumor histology alone (Winstead, 2023).

#### 3.3.6 Isocitrate dehydrogenase gene (*IDH*)

The *IDH1* and *IDH2* are frequently observed in various cancers, including gliomas and acute myeloid leukemia (AML). These mutations result in a gain of function, leading to the accumulation of the oncometabolite 2-hydroxyglutarate (2-HG), which contributes to tumorigenesis through various mechanisms including epigenetic alterations. Targeted therapies aimed at inhibiting mutant *IDH* enzymes, such as ivosidenib and enasidenib, have emerged as promising treatments for *IDH*-mutant cancers. These small molecule inhibitors specifically target mutant *IDH* enzymes, reducing 2-HG levels and restoring normal cellular function. Clinical trials have demonstrated efficacy in patients with relapsed or refractory AML and gliomas, leading to FDA approvals for these agents. As our understanding of the molecular mechanisms underlying *IDH*-mutant cancers continues to evolve, targeted therapies directed against mutant *IDH* enzymes hold significant promise for improving outcomes in patients with these malignancies.

A newly FDA-approved *IDH* inhibitor vorasidenib shows benefits for some low-grade gliomas in the human brain (NCI. *IDH*) (Winstead, 2023). The monotherapy of olutasidenib, an effective and specific inhibitor of *IDH1* mutation, has shown impressive and sustained remission rates, as well as significant outcomes like transfusion independence, among patients with relapsed or refractory (R/R) *IDH1*mut AML (Venugopal and Watts, 2023). This led to the approval olutasidenib for the treatment of *IDH1*mut AML.

#### 3.3.7 Human epidermal growth factor receptor 2 (HER2)

The agnostic landscape keeps evolving with the latest news of trastuzumab deruxtecan casting its bid for tissue-agnostic approval, underscoring the relentless quest for innovation (Jorgensen, 2023). FDA assigned priority review status to trastuzumab deruxtecan for the treatment of HER2-positive solid tumors in adults who have undergone prior treatment or have exhausted other options on 30 January 2024, and the approval was made on 5 April 2024. This marks the first time an antibody-drug conjugate (ADC) receives a tumor-agnostic indication, as well as the first approval for a HER2-targeted therapy with such a broad-spectrum indication. The indication would encompass patients with unresectable or metastatic HER2+ tumors with immunohistochemistry (IHC) 3+ status.

## 4 Discussion

Upon receiving pathological results for breast cancer patients, clinicians have been puzzled by the heterogeneity of specimens, by the variances and discrepancies in data interpretation, especially when encountering HER2 equivocality. Likewise, cfDNA methodological approaches that have been performed at different centers yielded cross-center data variances. Generally speaking, developing a well-accepted standard and operating procedures is important regardless of testing sites, whether this is being tested at a certified laboratory or in an in-house establishment. The reliability of genetic testing results is highly important because it initiates personalized care in precision medicine. A huge aftermath will emerge if the initial step is not guaranteed to be correct.

Real-world data indicated that genetic assays often discovered multiple gene alterations in one sample. Non-actionable alterations are not clinically meaningful to pursue. On the contrary, actionable alterations have data support utilizing certain specific therapies that target exactly these alterations or their cascading pathway. Medical complexity soon lies in scenarios of concurrent positivity of actionable alterations, where clinical decision-making could become challenging. Multiple factors should be considered before one mutation is determined of higher priority over the other. This is usually subject to a case-by-case analysis, which tends to compare efficacy data, safety profiling, and cost-effectiveness.

Clinical reality in implementing pharmacogenomic practice might be different between countries, except for seeing the same dataset presented at a scientific conference. This causes a dilemma in caring for international patients. Difficulties of fulfilling pharmacogenomic refinement not only lie in cultural and linguistic barriers but also extend to healthcare costs, data inconsistency, and governmental regulations.

There are nearly a dozen PD-1 inhibiting monoclonal antibodies commercially approved by the National Medical Products Administration (NMPA), China. These are yet to receive approvals from the FDA, despite being widely used to treat Chinese patients in place of expensive pembrolizumab (or nivolumab, etc.). The reason behind this is to relieve the payer’s burden or reduce healthcare costs. A good question to ask is: if an NMPA-approved PD-1 inhibitor is used on patients bearing MSI-H and/or high TMB, are we doing more harm than good when the clinical trials were conducted only on pembrolizumab? A time-consuming solution for this is to initiate a clinical trial of this PD-1 inhibitor in MSI-H patients. And that is what we expect.

When it comes to implementing pharmacogenomic practice, complexity follows if there is inconsistent testing result. Rebiopsy for a new molecular testing is one approach; for example, recurrent estrogen receptor-positive breast cancer patients might be treated with the recently approved elacestrant if they carry an *ESR1* (estrogen receptor 1 gene) mutation. A polygenic assessment approach is another option—mutations in phosphatidylinositol 3-kinase (PI3K), phosphatase and tensin homolog (*PTEN*), or protein kinase B (AKT) might lead the team to a different treatment pathway. There is no simple answer without a thorough consideration of other mutations and all other factors. Again, pharmacogenomic practice will be at a better level, when complemented with multidisciplinary efforts, in taking current challenges.

The current pharmacogenomic tool in hand provides exceptional assistance in personalized patient care. Some extra resources might offer a solution to overcome these difficulties. The most useful resource in the oncology world is the abundant availability of clinical research. A patient with *NTRK* fusion could be an ideal study participant for an ongoing repotrectinib investigational trial if repotrectinib has not been listed on the formulary. A patient who failed commercial *kRAS*-inhibiting treatments might seek to participate in a pan-*RAS* inhibiting clinical trial. With all these pharmacogenomic tactics and adequate research enrollments, precision medicine care will certainly step up to the next performing stage, and consequently benefit our medical community and patient care quality.

## 5 Conclusion

Pharmacogenomics and precision medicine continue to evolve rapidly, driven by advances in genomic sequencing technologies, molecular diagnostics, and targeted therapeutics. The integration of genomic information into clinical practice has transformed patient care, enabling the identification of actionable genetic alterations and the development of targeted treatment strategies tailored to individual patients. Tumor-agnostic approvals represent a paradigm shift in oncology, offering new opportunities for targeted therapy across diverse cancer types based on specific genetic biomarkers. Pharmacogenetic testing is of paramount importance in personalized medicine, guiding drug selection and dosing to optimize therapeutic outcomes while minimizing the risk of adverse drug reactions. As the field of precision medicine continues to expand, ongoing research and innovation will further refine our understanding of cancer biology and treatment response, paving the way for improved outcomes and better quality of life for patients with cancer.

## Abbreviations

2-HG, 2-hydroxyglutarate; 5-FU, 5-fluorouracil; ABCG2 Gene, ATP-Binding Cassette G2 subfamily; ADC, Antibody-drug conjugate; AKT, protein kinase B; AML, Acute myeloid leukemia; BCRP, breast cancer resistance protein; *BRAF*, Rapidly accelerated fibrosarcoma B-type gene; CYP, Cytochrome P450 (CYP); *DPD or DPyD*, Dihydropyrimidine dehydrogenase; *EGFR*, Epidermal Growth Factor Receptor; *ESR1*, estrogen receptor 1 gene; FDA, Food and Drug Administration; HNSCC, head and neck squamous cell carcinoma; HER2, Human Epidermal Growth Factor Receptor 2; *HRR*, homologous recombination repair; *HRD*, homologous recombination deficiency; HR, homologous recombination; IHC, Immunohistochemistry; *MET*, mesenchymal-epithelial transition factor; MDSCs, Myeloid-derived suppressor cells; *MGMT*, O-6-methylguanine-DNA methyltransferase; MMR, Mismatch repair; MRD, Minimal residual disease; *MSH6*, MutS Homolog 6; MSI, Microsatellite instability; mut/Mb, Mutations per Megabase; NER, nucleotide excision repair; NMPA, National Medical Products Administration, China; NCCN, National Comprehensive Cancer Network; NGS, Next-generation sequencing; *NTRK*, Neurotrophic tyrosine receptor kinase; PCR, Polymerase chain reaction; *PTEN*, phosphatase and tensin homolog; PI3K, phosphatidylinositol 3-kinase; *PMS2*, Postmeiotic segregation increased 2; *POLD1*, DNA polymerase delta 1; *POLE*, DNA polymerase epsilon; *RAS*, Rat Sarcoma Viral Oncogene Homolog; *KRAS*, Kirsten Rat Sarcoma Viral Oncogene Homolog; *RET*, Rearranged during transfection gene; *ROS1*, Receptor tyrosine kinase 1; Treg, regulatory T; TMB, Tumor mutational burden; TME, Tumor Microenvironment; TKIs, tyrosine kinase inhibitors; *TET2*, Ten Eleven Translocation 2; TPMT, Thiopurine S-methyltransferase; *UGT1A1*, Uridine diphosphate glucuronosyltransferase 1A1.
